# Bidirectional and Multi-User Telerehabilitation System: Clinical Effect on Balance, Functional Activity, and Satisfaction in Patients with Chronic Stroke Living in Long-Term Care Facilities

**DOI:** 10.3390/s140712451

**Published:** 2014-07-11

**Authors:** Kwan-Hwa Lin, Chin-Hsing Chen, You-Yin Chen, Wen-Tzeng Huang, Jin-Shin Lai, Shang-Ming Yu, Yuan-Jen Chang

**Affiliations:** 1 School and Graduate Institute of Physical Therapy, College of Medicine, National Taiwan University, Taipei 100, Taiwan; E-Mail: khlin@ntu.edu.tw; 2 Department of Physical Therapy, Tzu Chi University, Hualien 970, Taiwan; 3 Department of Management Information Systems, Central Taiwan University of Science and Technology, Taichung 406, Taiwan; E-Mail: chchen@ctust.edu.tw; 4 Institute of Biomedical Engineering, National Yang-Ming University, Taipei 112, Taiwan; E-Mail: d87921040@ntu.edu.tw; 5 Department of Computer Science and Information Engineering, Minghsin University of Science and Technology, Hsinchu 304, Taiwan; E-Mail: wthuang@must.edu.tw; 6 Department of Physical Medicine and Rehabilitation, College of Medicine, National Taiwan University and National Taiwan University Hospital, Taipei 100, Taiwan; E-Mail: jslai@ntu.edu.tw; 7 Department of Nursing, Central Taiwan University of Science and Technology, Taichung 406, Taiwan; E-Mail: smyu1946@ctust.edu.tw; 8 Institute of Biomedical Engineering and Materials Science, Central Taiwan University of Science and Technology, Taichung 406, Taiwan

**Keywords:** wireless sensor network, telerehabilitation, stroke, balance

## Abstract

**Background:**

The application of internet technology for telerehabilitation in patients with stroke has developed rapidly.

**Objective:**

The current study aimed to evaluate the effect of a bidirectional and multi-user telerehabilitation system on balance and satisfaction in patients with chronic stroke living in long-term care facilities (LTCFs).

**Method:**

This pilot study used a multi-site, blocked randomization design. Twenty-four participants from three LTCFs were recruited, and the participants were randomly assigned into the telerehabilitation (Tele) and conventional therapy (Conv) groups within each LTCF. Tele group received telerehabilitation but the Conv group received conventional therapy with two persons in each group for three sessions per week and for four weeks. The outcome measures included Berg Balance Scale (BBS), Barthel Index (BI), and the telerehabilitation satisfaction of the participants.

**Setting:**

A telerehabilitation system included “therapist end” in a laboratory, and the “client end” in LTCFs. The conventional therapy was conducted in LTCFs.

**Results:**

Training programs conducted for both the Tele and Conv groups showed significant effects within groups on the participant BBS as well as the total and self-care scores of BI. No significant difference between groups could be demonstrated. The satisfaction of participants between the Tele and the Conv groups also did not show significant difference.

**Conclusions:**

This pilot study indicated that the multi-user telerehabilitation program is feasible for improving the balance and functional activity similar to conventional therapy in patients with chronic stroke living in LTCFs.

## 1. Introduction

Balance ability is impaired in the majority of patients following a stroke [1]. Previous studies indicated that balance performance could be improved following either face to face conventional balance training or group therapy for participants with sub-acute or chronic stroke [2]. Lai *et al.* applied eight-week videoconferencing for community-dwelling post-stroke subjects and the result demonstrated a significant improvement in the Berg Balance Scale (BBS) scores, although no conventional training or control group was compared [3]. The study of Cikajlo *et al.* further indicated that the use of telerehabilitation with virtual reality-supported balance training could have similar effects as the conventional balance training group on balance in patients with sub-acute to chronic post-stroke condition living in rehabilitation hospitals [4]. Approximately 10% to 30% of stroke survivors in England are discharged to nursing homes or long-term care facilities (LTCFs) [5,6]. To date, only a few studies have investigated the effect of balance training in post-stroke subjects living in LTCFs, and those studies did not include conventional training groups [7].

Telecommunication tools for remote and home health management have been developed through the years [8–13]. Wireless sensor network (WSN) technologies with the aid of information communication technology (ICT) can be used for the transmission and monitoring of the vital signs of clients to physicians or therapists [11–15]. However, the results about cost-effectiveness of tele-care are controversial [16]. Furthermore, introducing a telerehabilitation system without adequate monitoring and evaluation functions can be risky for decision making [17]. Our previous studies have indicated that the needs and satisfaction of the users must also be considered before implementing a healthcare system [14,15].

The advantages of telerehabilition for patients with neurological diseases include increased access to post-acute rehabilitation services at long distance [18,19], and less cost than in-patient services [7,20,21]. Post-stroke patients with functional disability will need long term rehabilitation care [22]. Comparing to individual therapy, the short-term group psychotherapy is a cost-effective treatment strategy to reduce the cost of manpower for older adults living in LTCFs [23], as well as for stroke patients in rehabilitation units [24]. Hence, the current study aimed to develop a multi-user telerehabilitation system (*i.e.*, group therapy) with emphasis on balance training for patients living in LTCFs.

The effect of telerehabilitation on balance might be different from the conventional therapy in chronic stroke patients. The research hypotheses in this study would be: (1) significant improvement on balance and functional activity after telerehabilitation; (2) significant differences between telerehabilitaion and conventional training groups on balance, functional activity, and satisfaction in patients with chronic stroke living in LTCFs.

## 2. Experimental Section

### 2.1. Telerehabilitation Systems

**Figure 1.**
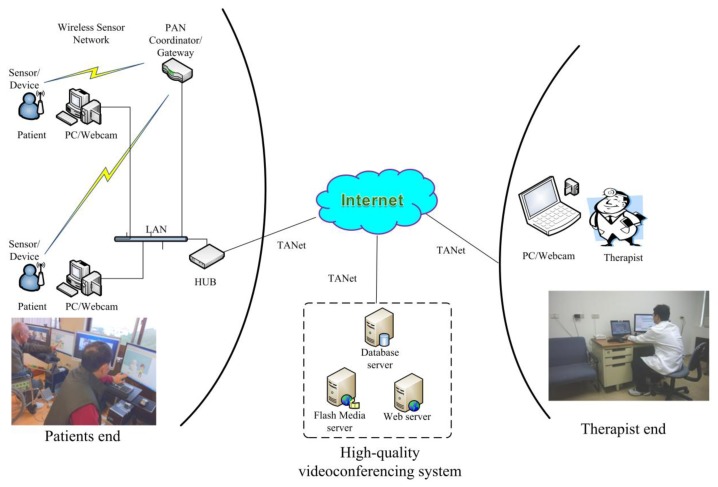
Architecture of the designed WSN telerehabilitation system.

A WSN telerehabilitation system, including “therapist end”, “client end”, and a data center, was designed and established (Figure 1). The telerehabilitation systems at the “therapist end” (*i.e.*, research laboratory at National Taiwan University in Taipei, Taiwan) for videoconferencing included a notebook computer with a PCI network card, a Logitech webcam, a cross shaped sound recorder (VoIP conference station, Taiwan), and a switch hub. The video and audio signals of the physician are sent to the “client end' through the Taiwan Academic Network (TANET). The telerehabilitation systems at the “client end” (*i.e.*, LTCF) included a personal computer with a PCI network card, two 55.88 cm screens (one regular screen for video communications and one touch screen for interactive games), and a Logitech webcam.

The online telerehabilitation functions in this pilot study [25] included: (1) live video conferencing function in the “Online Rehabilitation” section; (2) “Rehabilitation Education and Consultation Functions” section; and (3) “Assessment and Therapy Functions” section. The high quality video conferencing system is implemented using Adobe^®^ Media Server (Adobe^®^ Systems Software Ireland Ltd., San Jose, CA, USA). A customized integration information window of the video and vital signs of the two client users can be shown on the screen of the therapist. Meanwhile, the live video of the therapist is shown on each screen of the client users, enabling them to follow the instruction of the therapist at the same time.

The vital signs monitor was set at the official website. Through the aid of the information communication technology (ICT), the therapist can understand and monitor the progress of diseases, thereby effectively supervising the situation. The vital signs of heart rate (HR), oxygen saturation (SpO_2_), and blood pressure (BP) are measured online using the pulse oximeter [Ninon Medical, Plymouth, MN, USA] and blood pressure monitor sensors (Clever Chek, TD-3250, Taidoc Technology Corp., New Taipei City, Taiwan], respectively, with the ZigBee wireless designed by a team member (*i.e.*, Professor Huang). The detailed design can be found in previous research [14]. The normal ranges were set in the software to give precautious signal when the responses of users are out of normal range. The alert would start when the responses of users during the rehabilitation program are in the contra-indication range (*i.e.*, HR > 140 b/min, SpO_2_ < 90%, SBP/DBP > 160/110). The therapist can control the vital sign sensors remotely using bidirectional sensor control technology; that is, turning the sensor into active mode or sleep mode [15]. At the first visit, one volunteer or nonmedical person was instructed by the therapist about putting on and off the measuring device, such as arm cuff for blood pressure and fingertip for heart rate monitor; hence, he or she could assist the user as necessary.

### 2.2. Study Protocol

#### 2.2.1. Study Design

This pilot study was a multi-sites, blocked randomization design (*i.e.*, one kind of randomization) [26,27], and the participants in each LTCF were randomly assigned to the telerehabilitation (Tele) as the experimental group and the conventional therapy group (Conv) as the control group. The randomization was performed by random numbers which were generated by computer.

#### 2.2.2. Recruitment of Participants

The participants were recruited from three different LTCFs (two rural facilities in Taipei, Taiwan and one rural facility in Taichung, Taiwan). Three LTCFs located at different distances allowed us to examine the telerehabiliation availability with Taiwan wired network (TANET) at short (7.4 km), medium (40 km), and long distances (175 km). The inclusion criteria for participants were: (1) a history of cerebral vascular accident (including first and recurrent stroke) for more than six months; (2) living in LTCFs for more than three months; (3) having active movement of the proximal part of upper extremity in the hemiparetic side (Brunnstrom stage U/E ≥ 3) [28]; (4) being able to sit for short periods without hand support for at least 30 s; (5) having cognitive status screened using the Mini-Cog test [29] and being able to follow the instruction; and (6) being able to communicate and follow a three-step command. Meanwhile, the exclusion criteria were: (1) having other neuromusculoskeletal condition and systemic diseases, such as Parkinson's disease and uncontrolled heart disease; (2) blindness and deafness; and (3) having a psychiatric history.

**Figure 2.**
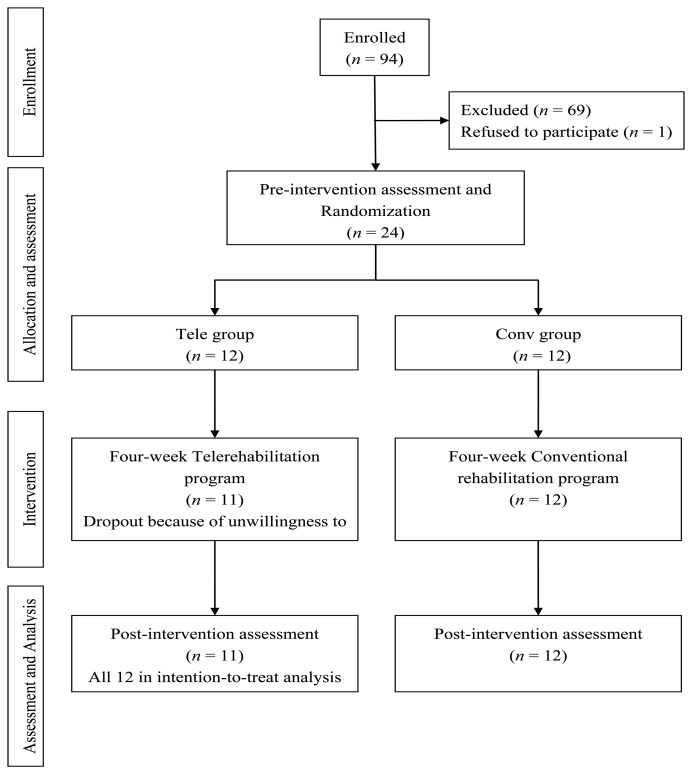
Flow diagram of experimental procedures.

The rater was blinded to the allocation of participants. A total of 94 post-stroke participants were screened by a therapist with face-to-face interviews, and 69 participants were excluded because they did not meet the inclusion criteria or were unwilling to participate in the project (Figure 2). Twenty-four participants completed the training, with eight participants in each facility. All participants were informed on ethical approval and consequently signed the consent form, which was reviewed and approved by the Human Subjects Review Committee at National Taiwan University Hospital, Taiwan. Given that this was a pilot study, the sample size was not calculated. Protocol records were made available to the public through the ClinicalTrials.gov website. Ethics approval was obtained from the National Taiwan University Hospital.

#### 2.2.3. Intervention Procedures

The treatment program for both groups included three sessions of training per week for four weeks, with the duration of approximately 50 min for each session. The therapist instructed standing balance training from easy to difficult, depending on the severity and recovery of the participants (Appendix A, Table A1). The tele-balance training focused on 10 min of standing exercise according to 3D animation exercise videos which were Maya/3D Max systems (Appendix B), and about 10 min of 3D interactive games with finger touching the touch screen in standing posture (Appendix C). The therapist could monitor the sequence and duration with light to moderate exercise intensity (Borg scale 12–14). The therapist can instruct both participants in a group to do similar programs as much as possible and allow them to play ball together during the balance training.

One physical therapist performed the pre- and post-assessments for both groups and blinded to the assignment. One therapist conducted the telerehabilitation balance training at the “therapist end” (*i.e.*, the medical center of Medical College, National Taiwan University) to each facility for one month, separately. One volunteer or nonmedical person was assigned at the “patient end” for safety and assistance in telerehabilitation and conventional training. Three experienced therapists conducted conventional balance training (*i.e.*, one therapist for each facility), who were trained to conduct similar programs.

#### 2.2.4. Telerehabilitation Programs

As shown in Appendix A, Table 1, three components were monitored: (1) changing body position: from sitting to standing and from static to dynamic; (2) changing environment: from firm to foam seat; and (3) using upper extremities: from no manipulation to arm-hand manipulation on the touch screen or holding the small ball. Two screens were set at the “therapist end”; hence, the therapist could see each participant either on the same screen or on a separate screen at the same time, depending on the opened window size of the participant.

#### 2.2.5. Conventional Training Programs

Two post-stroke participants attended the same session as the small therapy group. The therapist conducted conventional balance training programs following simple to comples principle (Appendix A, Table A1). However, the small ball and peg bars are used for hand manipulation during sitting and standing balance training.

### 2.3. Outcome Measures

The Berg Balance Scale (BBS) and Barthel Index (BI) scores of the participants were assessed by a therapist for balance and functional activity, respectively. The satisfaction of participants was also assessed through their answers on the questionnaires. The BBS with fourteen items (including sitting and standing balance) was used for balance evaluation, and the score ranged from 0 to 56, with a higher score for better balance [30].

The BI was used to evaluate daily functional activities of the participants. The index included self-care, such as personal hygiene, (subscore: 40) and mobility domains, such as 50-yard walking and going up and down the stairs, (subscore: 60), with the total score ranging from 0 to 100 [31]. A higher score indicates less severity of stroke.

A questionnaire survey was conducted to understand the satisfactions and attitudes of the participants toward using telerehabilitation system. The questionnaire was derived from successes of Technology Acceptance Model and Model of information systems employed by David [32] and DeLone and McLean [33], which were developed and shown to have a high degree of convergent and discriminate validity. In the current study, the satisfaction questionnaire for Tele group included: (1) System Environment Satisfaction: satisfaction of rehabilitation environment and devices; (2) Perceived Usefulness: usefulness of the telerehabilitaion system; (3) Perceived Ease of Use: ease use of the telerehabilitaion system; (4) Attitude Toward Using: intent to use the telerehabilitation system; and (5) Perceived Satisfaction of System: satisfaction of the rehabilitation process. The satisfaction questionnaire for Conv group only included item 1 and item 5. Each item was measured with a Likert scale ranging from 1 (strongly disagree) to 5 (strongly agree). The values of each dimension were derived from the average scores of the relative item scores of each dimension.

### 2.4. Data and Statistical Analysis

SPSS version 17.0 for Windows was used for data analysis. Analyses of intention to treat were used for one drop-out. The normal distribution of the data was analyzed by Kolmogorov-Smirnov test (KS-test). Demographic data were analyzed by a chi-square test (for nominal data) and t-test (for interval data). The data of BBS and BI scores were normally distributed; hence, the parametric analysis was used. The group and individual effects of treatment (2 × 2) were analyzed using combined two-way ANOVA with post hoc Bonferroni analysis. *p* < 0.05 was considered statistically significant. However, the questionnaire with the small sample size, the non-parametric statistics (Mann-Whitney U-test, and Wilcoxon Signed-Rank test) was used [34]. *p* < 0.05 was set as statistically significant.

## 3. Results

Details of the demographic data are shown in Table 1. No significant differences were found for age, height, weight, and severity (Brunnstrom stage) between the Tele and the Conv groups.

### 3.1. Balance and Functional Activity

Both the Tele and Conv groups exhibited significant training effect on BBS (*p* < 0.001) score; however, no significant difference was observed between the two groups (Table 2). Significant training effects on total and self-care score of BI and basic daily activity were also observed in both groups; however, no significant difference was observed between the two groups (Table 2). No significant change in mobility was noted (*p* = 0.088), which included 50-yard walking and going up and down the stairs.

### 3.2. User Satisfaction

The questionnaires were distributed to the participants at the post-intervention assessment period. Missing data were found on questionnaires from one patient in the Tele group and three patients in the Conv group. Thus, this study comprised questionnaires of 11 patients in the Tele group and nine patients in the Conv group. Non-parametric Mann-Whitney U-test was used.

**Figure 3.**
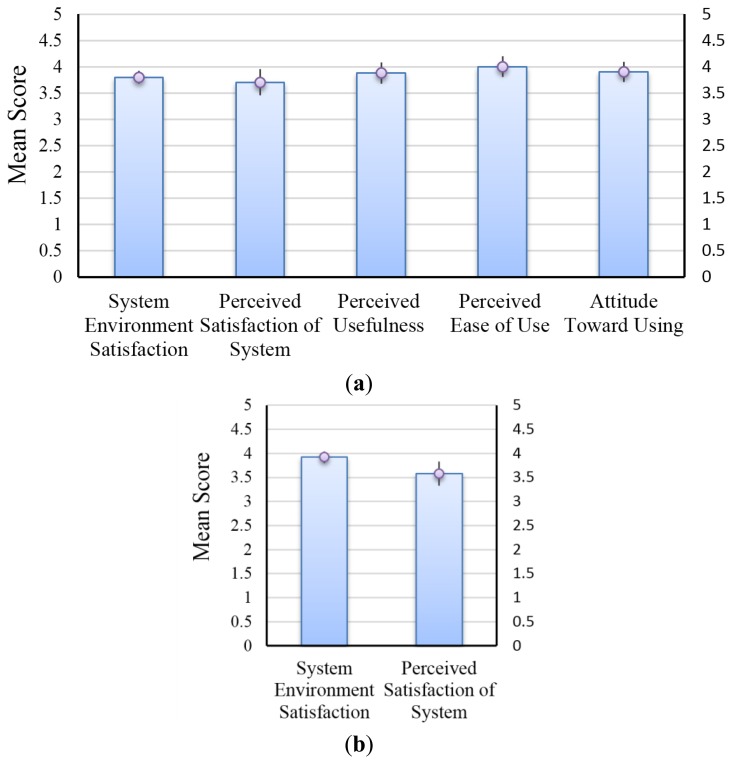
Mean participant responses of (**a**) telerehabilitation group (Tele); and (**b**) conventional group (Conv) to the satisfaction questionnaire.

Overall, the participants of the Tele group (Figure 3a) and Conv group (Figure 3b) indicated good level of satisfaction with all questions. The Conv group performed questions of two dimensions, *i.e.*, System Environment Satisfaction and Perceived Satisfaction of System. Except these two dimensions, the Tele group performed questions of three more dimensions, *i.e.*, Perceived Usefulness, Perceived Ease of Use and Attitude Toward Using. The Wilcoxon Signed-Rank test was used to determine the significance of the differences between the score on each dimension and score 3 (Figure 3). Most participants perceived ease of use and high attitude toward using the telerehabilitation system or willingness to recommend the system to other patients (*p* < 0.05 and median values are larger than 3). The results showed no significant difference on all the items, including Perceived Usefulness (*p* = 0.053) and Perceived Satisfaction of System (*p* = 0.052) (Figure 3a). Analyzed data within the Tele group showed significant difference on Perceived Usefulness (*p* = 0.016) and Perceived Satisfaction of System (*p* = 0.049) (Figure 3a). In addition, results showed that the response of the participant on System Environment Satisfaction and Perceived Satisfaction of System did not show significant difference between the two groups (Figure 3b).

## 4. Discussion

The use of a bidirectional and multi-user telerehabilitation system in rehabilitation programs is feasible to improve balance and functional activity of residents with chronic hemiplegia in LTCFs. However, no significant differences in balance and functional activity were demonstrated between the intervention groups compared with conventional therapy group. The characteristics of the present telerehabilitation program included: (1) a short-term small theraphy group, and (2) using the commercially inexpensive touch screen with 3D interactive games for the somatosensory feedback of hand [35], as well as the cognitive task (*i.e.*, game play) during sitting and standing balance training [36].

In the current study, the mean reported differences (pre *vs.* post) of Berg Balance Scale in both groups were approximately 4 to 5. However, the study of Hiengkaew *et al.* [37] found that the minimal clinically important difference (MCID) of Berg Balance Scale in chronic stroke is 4.66. Thus, both the telerehabilitation and conventional therapy could maintain or slightly improve balance in the small sample size of participants with chronic stroke living in an LTCF. Given that longitudinal studies have shown that almost all stroke patients experience slight predictable degrees of functional recovery in the first 6 months post-stroke, the present study would be less confounded by natural recovery [38]. However, the telerehabilitation system used in this study would require further studies with a larger sample size.

A significant improvement of self-care in both the Tele and Conv groups was observed (Table 2). The upper extremity function in eating, dressing, and so on increased slightly in both the Tele and Conv groups. Interestingly, upper extremity manipulation exercises in training would have similar effect through either touch screen game play (Tele group) or ball/peg bar play (Conv group). Similar programs with eye-hand coordination training also showed an important effect in daily function activity of the participants [7,35]. The possible reasons for similar training effects beween telerehabilitation and conventional training might be due to: (1) similar training duration and frequency; (2) similar progression strategy that being from simple to complex and from static to dynamic; (3) similar principles of feedback (*i.e.*, knowledge of performance and knowledge of result) to correct balance, although telerehabilitation emphasized verbal/visual feedback from remote therapist, but conventional therapy emphasized verbal/hepatic feedback from the nearby therapist [18,35].

Data on the attitude of participants toward telerehabilitation system usage was provided. However, no significant difference was found on all the items. Data within the Tele group analyzed using Wilcoxon Signed-Rank Test to examine the difference between Perceived Usefulness and average value (*i.e.*, 3), as well as between Perceived Satisfaction of System and value 3, showed significant difference on Perceived Usefulness and Perceived Satisfaction of System. Participants with high positive attitude toward rehabilitation would consider telerehabilitation system more useful and easy to use. Given the reduced round trip time from the institution to the hospital and the intensive interest in game playing, participants in the Tele group have greater intention of recommending the telerehabilitation system to other patients.

### 4.1. Limitations

The limitations of the present work include the following. First, hemiplegic patients who participated in the Tele and Conv groups suffered mild to moderate stroke without cognitive impairment. Thus, the results may not be applicable to patients with severe cases. Second, being a high-technology web-based telerehabilitation, the operational technique training of the user, family members and the therapist was required. Finally, the financial requirement for computer devices and internet service fees may have to concern for possible participants living in LTCFs.

### 4.2. Clinical Application

The reduced cost of telerehabilitation for the elderly in LTCFs has been previously published [39], and most participants were satisfied with the program. The present study provides the information related to the feasibility and effect of telerehabilitation on patients with chronic stroke in LTCFs. To reduce the frequent long distance travel of therapists or patients for care services [40], telerehabilitation is suggested to be set up in LTCFs, especially in rural areas, for patients with mild-to-moderate impairment.

The novel bidirectional sensor control function implemented in this study also has advantages for clinical use. The sleep mode function of the sensors of the device can help save battery power. The physical therapist can easily obtain the vital signs of the patients by turning the sensors into active mode anytime and anywhere through the use of a web browser. This advantage reduces the threshold of patients and caregivers accessing the telerehabilitation system. Hence, the score on perceived satisfaction of the system reached as high as 3.9.

Another important function of the present telerehabilitation system is the provision of one therapist for two patients at the same time. In reality, given a capable hardware, the system can provide multiple patients connecting to the server and performing rehabilitation programs at the same time. Furthermore, the required manpower for conducting telerehabilitation programs is reduced. Vital signs and videos of each patient can also be stored independently during the telerehabilitation program.

## 5. Conclusions

The use of bidirectional and multi-user telerehabilitation systems in rehabilitation programs is feasible for improving balance and self-care functions in patients with chronic stroke living in LTCFs. Although the present telerehabilitation system is not superior to conventional balance training, the results indicated that communication between stroke patients in long term care facility and the therapist can be much satisfactory through the Internet system. Telerehabilitation with small therapy group can be an alternative or adjunct therapy for patients with mild to moderate stroke living in LTCFs.

## Figures and Tables

**Table 1.** Demographic data of participants in the telerehabilitation (Tele) and conventional balance training (Conv) groups.

**Tele**					**Conv**			

**Name of Institution**	**CT**	**SL**	**HC**	***Total***	**CT**	**SL**	**HC**	***Total***
Gender (F/M)	2/2	0/4	0/4	***2/10***	0/4	2/2	3/1	***5/7***
Affect side (L/R)	1/3	0/4	2/2	***3/9***	3/1	1/3	4/0	***8/4***
Age	78.8(5.2)	79.3(5.9)	65.8(2.2)	***74.6 (2.3)***	76.0(10.1)	81.5(11.2)	69.3(13.6)	***75.6 (3.4)***
Body height	159.0(9.0)	168.3(3.3)	166.8(4.7)	***164.7 (2.0)***	160.0(6.4)	160.8(12.7)	156.3(2.5)	***159.0 (2.3)***
Body weight	57.0(25.9)	71.3(14.8)	60.3(10.1)	***62.8 (5.1)***	67.3(6.6)	60.0(12.3)	63.0(7.4)	***63.4 (2.5)***
Duration	21.3(8.3)	18.8(9.8)	55.5(30.9)	***31.8 (11.3)***	49.8(22.7)	40.3(26.8)	35.0(20.3)	***41.7 (12.4)***
Brunnstrom stage	5.0 (0.4)	6.0 (0.0)	3.3 (0.3)	***4.8 (0.4)***	5.0 (0.7)	6.0 (0.0)	3.3 (0.3)	***4.8 (0.4)***
UE_Proximal	5.0 (0.4)	6.0 (0.0)	3.0 (0.4)	***4.7 (0.4)***	5.0 (0.7)	5.8 (0.3)	3.0 (0.4)	***4.6 (0.4)***
UE_Distal LE	5.0 (0.4)	6.0 (0.0)	4.0 (0.4)	***5.0 (0.3)***	5.0 (0.7)	5.8 (0.3)	3.5 (0.5)	***4.8 (0.4)***

CT: Cardinal Tien-Nursing Home; SL: Suang-Lien Elderly Center-Nursing Home; HC: Hui-Chun Nursing Home; UE: Upper extremity; LE: lower extremity. Data were expressed as means (SE). Significant level is *p* < 0.05.

**Table 2.** Effects of intervention on balance and functional activity in telerehabilitation (Tele) and conventional balance training (Conv) groups.

	**Tele (*n* = 12) Pre Post**		**Conv (*n* = 12) Pre Post**		**P_T_**	**P_G_**	P_T*G_
**Berg Balance Scale**	20.4 (4.9)	24.6 (5.3)	22.4 (5.3)	26.9 (5.2)	<0.001 *	0.770	0.829
**Barthel Index**	52.9 (9.5)	57.9 (0.9)	57.9 (7.7)	60.8 (6.5)	0.008 *	0.739	0.451
**Self-care**	36.7 (5.4)	40.0 (5.0)	39.2 (5.3)	41.3 (4.2)	0.014 *	0.791	0.543
**Mobility**	16.3 (4.6)	17.9 (4.5)	18.8 (3.2)	19.6 (3.2)	0.088	0.710	0.557

Data were expressed as means (SE). Two-way Mixed ANOVA; P_T_: Training main effect; P_G_: Group main effect; P_T*G_: Training and group interaction. Data were analyzed by two-way mixed ANOVA; * *p* < 0.05.

**Figure B1.**
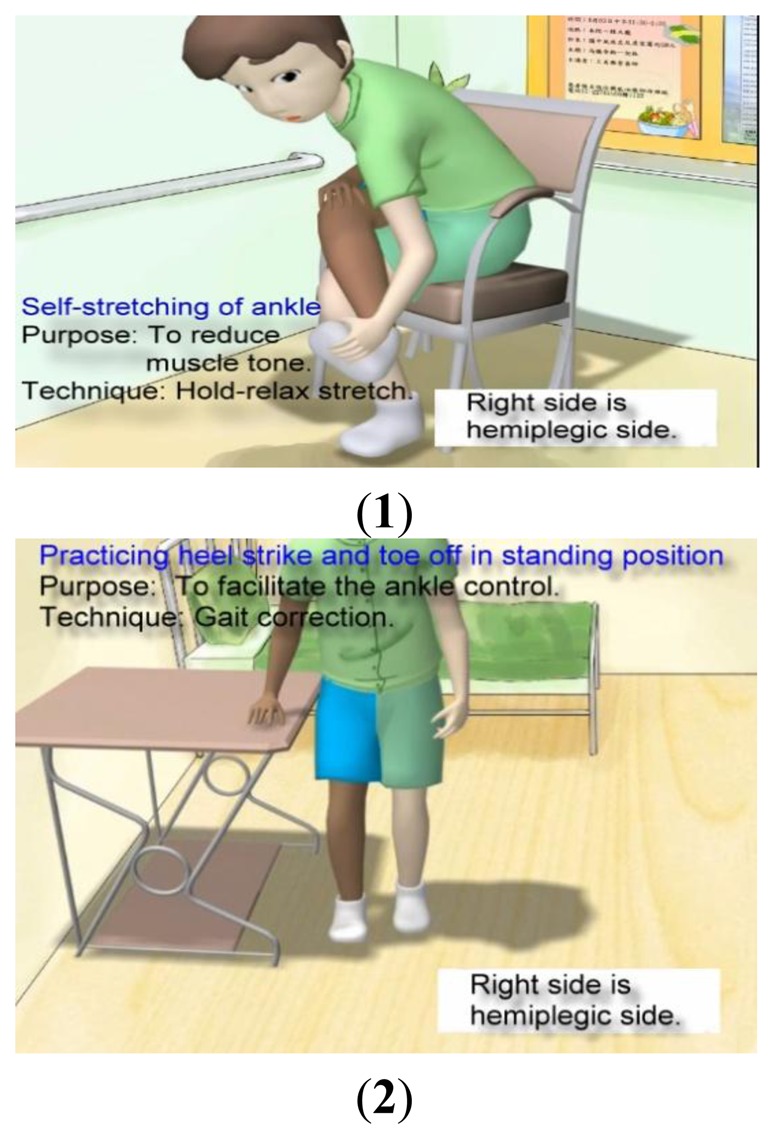

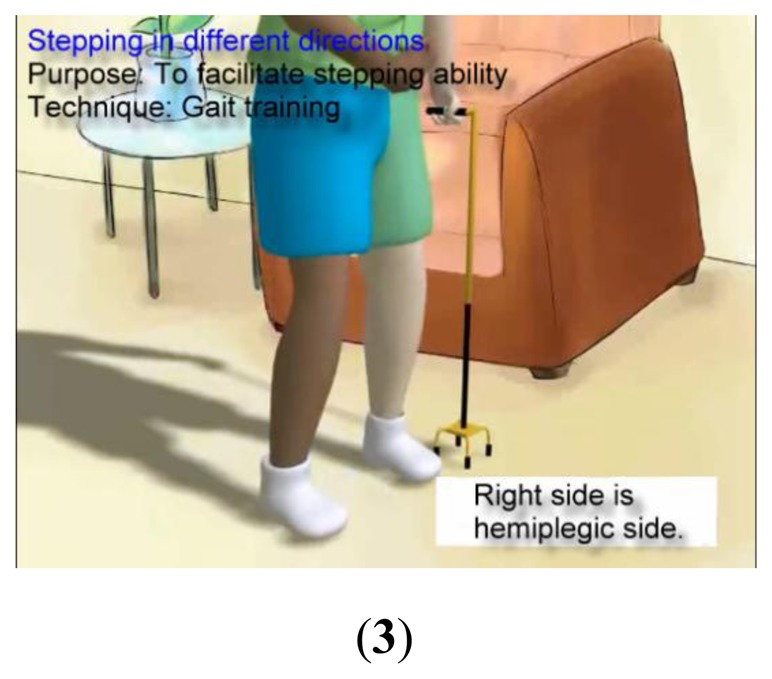
The 3D animation exercise videos for hemiplegic patients*. (Dark color: Hemiplegic side); (**1**) Self-stretching of ankle; (**2**) Practicing heel strike and toe off in standing position; (**3**) Stepping in different directions.

**Figure C1.**
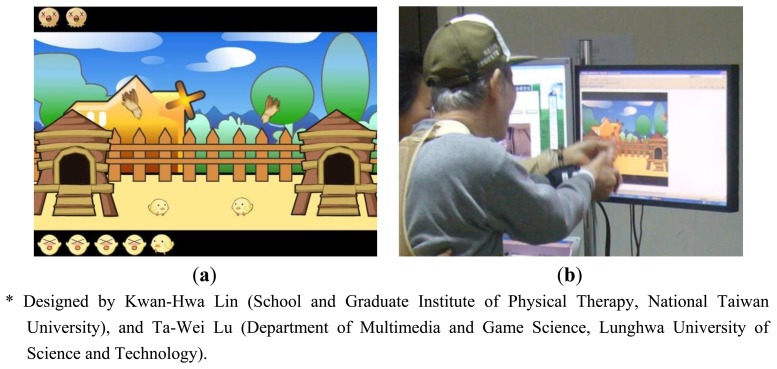
Example of 3D animation interactive games with touch screen control*.

## Appendix A

**Table A1.** General principle of programs for progression.

Tele Group (Visual and verbal instructions by a remote therapist via computer and screen monitor)		Conv Group (Visual and verbal instructions by a nearby therapist via personal face-to-face contact)
Phase 1	Static/dynamic sitting balance on firm surface Touch screen manipulation Getting acquainted with partner	Static/dynamic sitting balance on firm surface Ball manipulation Getting acquainted with partner
Phase 2	Static/dynamic sitting balance on foam Touch screen manipulation (increasing speed) Simple interaction with partner	Static/dynamic sitting balance on foam Ball manipulation or placing peg bars Simple interaction with partner
Phase 3	Static standing balance on firm surface Touch screen manipulation during sitting (increasing difficulty) Transfer ball to partner while sitting	Static standing balance on firm surface Ball manipulation or placing peg bars while sitting. Transfer ball to partner while sitting
Phase 4	Dynamic standing balance on firm surface Touch screen manipulation while sitting or standing Transfer ball to partner while standing	Dynamic standing balance on firm surface Ball manipulation or placing peg bars while standing Transfer ball to partner while standing

The progression will depend on the condition of the participant. Standby supervision or assistance is provided by volunteers or non-medical personnel.
